# Left Atrium by Echocardiography in Clinical Practice: From Conventional Methods to New Echocardiographic Techniques

**DOI:** 10.1155/2014/451042

**Published:** 2014-06-09

**Authors:** Roberta Ancona, Salvatore Comenale Pinto, Pio Caso, Antonello D'Andrea, Giovanni Di Salvo, Fortunato Arenga, Maria Gabriella Coppola, Vincenzo Sellitto, Maria Macrino, Raffaele Calabrò

**Affiliations:** ^1^Noninvasive Cardiology Unit, Department of Cardiology, Monaldi Hospital, A.O.R.N. dei Colli, Via Leonardo Bianchi, 80131 Naples, Italy; ^2^Monaldi Hospital, A.O.R.N. dei Colli, Via Leonardo Bianchi, 80131 Naples, Italy

## Abstract

Although often referred to as “the forgotten chamber”, compared with left ventricle (LV), especially in the past years, the left atrium (LA) plays a critical role in the clinical expression and prognosis of patients with heart and cerebrovascular disease, as demonstrated by several studies. Echocardiographers initially focused on early detection of atrial geometrical abnormalities through monodimensional atrial diameter quantification and then bidimensional (2D) areas and volume estimation. Now, together with conventional echocardiographic parameters, new echocardiographic techniques, such as strain Doppler, 2D speckle tracking and three-dimensional (3D) echocardiography, allow assessing early LA dysfunction and they all play a fundamental role to detect early functional remodelling before anatomical alterations occur. LA dysfunction and its important prognostic implications may be detected sooner by LA strain than by volumetric measurements.

## 1. Introduction

Clinical evaluation of the left atrium (LA) is important in many cardiac and noncardiac diseases, requiring an in-depth understanding of anatomy and physiology. The systematic assessment of LA function is not uniformly carried out. This is partly due to the enormous attention given to the evaluation of the left ventricle (LV), a lack of familiarity with ultrasound techniques that can be used in imaging the LA, and the absence of validation of a unique standardized technique to investigate LA deformation. Until recently the LA had been subordinated to the LV, but cardiologists now recognize that LA function is indispensable to normal circulatory performance, conditioning the morbidity and mortality in several diseases. So an early detection of LA dysfunction is anticipated to provide new insight into pathophysiology and clinical management of several conditions such as atrial fibrillation (AF), valvular heart disease, hypertension, heart failure (HF), and cardiomyopathy. Echocardiography is therefore the imaging modality of choice for screening and serially following patients with diseases involving the LA morphology and function [1]. Nowadays, by new echocardiographic techniques, such as strain (S) Doppler, speckle tracking, and 3D echocardiography, we are able to early recognize atrial dysfunction, before clinical manifestations and earlier than standard echocardiographic parameters [2–8].

## 2. Anatomy

The LA is located in the mediastinum, oriented leftward and posterior to the right atrium (RA). LA structure is characterized by a pulmonary venous component, a lateral finger-like appendage, an inferior vestibular component, which surrounds the mitral valve orifice, and a prominent body that shares the septum with the RA. The pulmonary venous component with venous orifices at each corner is situated posteriorly and superiorly, and directly confluent with the body. The walls of the LA can be described as superior (roof), posterior (inferoposterior), left lateral, septal, and anterior. The majority of the atrium is relatively smooth, whereas the appendage is rough with pectinate muscles. The walls are composed of one or more overlapping layers of differently aligned myocardial fibres, with marked regional variations in thickness. Circular fibres are more or less parallel to the atrioventricular valve plane, whereas longitudinal fibres run nearly perpendicularly. Oblique fibres are those inclined between the two major axes [9].

## 3. Standard Echocardiographic Methods

### 3.1. LA Dimensions

Increased LA size is associated with adverse cardiovascular outcomes [10, 11]. LA size correlates with both LA and LV functions, and it is a strong predictor of cardiovascular death and morbidity. Relationships exist between increased LA size and the incidence of AF and stroke, risk of overall mortality after myocardial infarction, and risk of death and hospitalization in patients with dilated cardiomyopathy [12–15]. LA is a marker of both the severity and chronicity of diastolic dysfunction and magnitude of LA pressure elevation [10].

**Figure 1:**
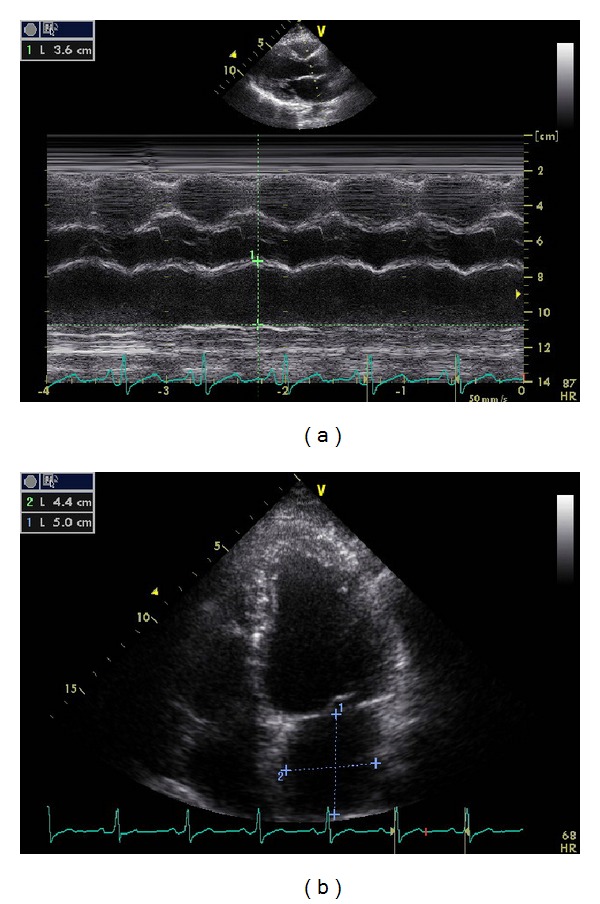
LA dimensions: anteroposterior diameter in parasternal long-axis view (a); longitudinal and transverse diameters in 4-chamber view (b).

The LA size is measured at the end-ventricular systole when the LA chamber is at its greatest dimension, in long-axis view (anterior-posterior diameter) and in 4-chamber view (longitudinal and transverse diameters) [16] (Figure 1).

Although linear measurements have been shown to correlate with angiographic measurements and have been widely used in clinical practice and research, they inaccurately represent true LA size, given that the LA is not a symmetrically shaped 3D structure.

Evaluation of the LA in the AP dimension assumes that a consistent relationship is maintained between the AP dimension and all other LA dimensions as the atrium enlarges, which is often not the case [17]. Expansion of the LA in the AP dimension may be constrained by the thoracic cavity between the sternum and the spine. Predominant enlargement in the superior-inferior and medial-lateral dimensions will alter LA geometry such that the AP dimension may not be representative of LA size, and it should be accompanied by LA volume determination in both clinical practice and research [16–18].

### 3.2. LA Volumes

Because the LA is an asymmetrical cavity, LA size is more accurately reflected by a measurement of volume rather than area or linear dimension. So in clinical practice, volume determinations are preferred over linear dimensions; in addition, the strength of the relationship between cardiovascular diseases is stronger for LA volume than for LA linear dimensions [19, 20].

Conventional echocardiography allows measurement of all LA volumes [16].

**Figure 2:**
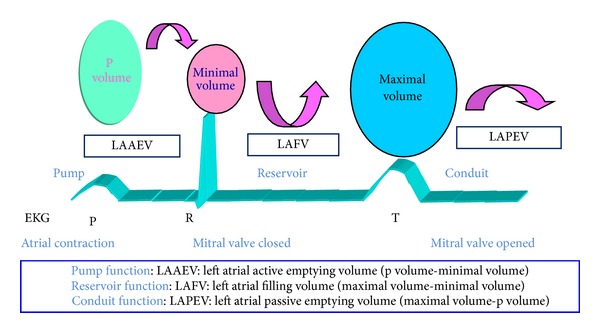
Three different atrial volumes during cardiac cycle.

LA passive volumes consist of preatrial contraction volume (*V*_*p*_), measured at the onset of the P-wave on an electrocardiogram; minimal LA volume (*V*_min⁡_), measured at the closure of the mitral valve in end-diastole; and maximal LA volume (*V*_max⁡_), measured just before the opening of the mitral valve in end-systole. LA active volumes are LA reservoir volume or LA filling volume (LAFV) (*V*_max⁡_ -*V*_min⁡_); LA conduit volume or LA passive emptying volume (*V*_max⁡_ -*V*_*p*_); and LA contractile volume (*V*_*p*_ -*V*_min⁡_) [16] (Figure 2).

The difference between maximum and minimum LA volume divided by the minimum LA volume is used as index of atrial compliance [4, 5].

**Figure 3:**
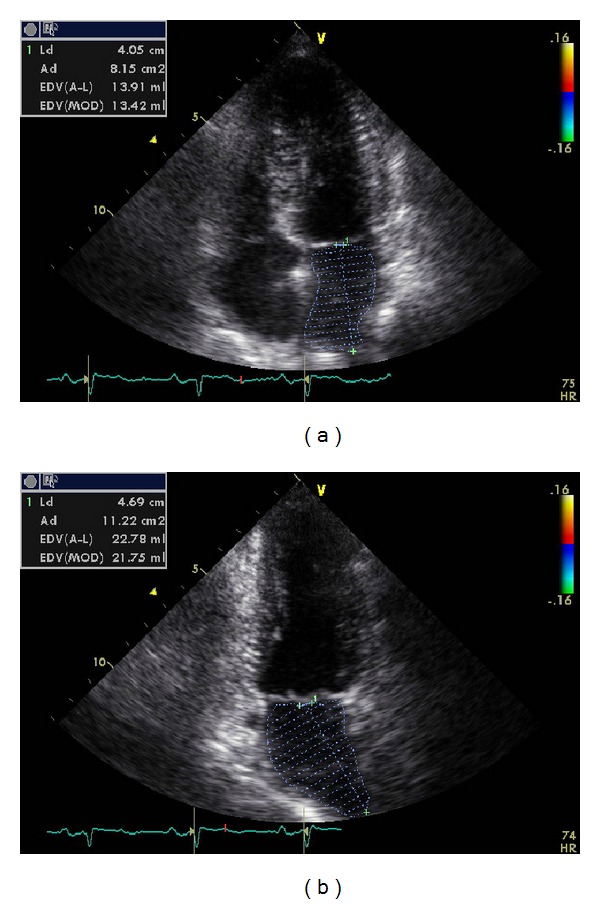
Measurement of left atrial volume from biplane method of disks (modified Simpson's rule) using apical 4-chamber (a) and apical 2-chamber (b) views at ventricular end-systole (maximum volume).

The American Society of Echocardiography and the European Association of Echocardiography recommend the measurement of LA volumes by ellipsoid model and Simpson's method in four- and two-chamber apical views [16] (Figure 3). Volume determined using linear dimensions is very dependent on careful selection of the location and direction of the minor-axis dimensions and has been shown to significantly underestimate LA volume. The use of Simpson's method in this way requires the input of biplane LA planimetry to derive the diameters. Optimal contours should be obtained orthogonally around the long axis of the LA using transthoracic echocardiography apical views. Care should be taken to exclude the pulmonary veins and LA appendage from the LA tracing. The inferior border should be represented by the plane of the mitral annulus [16]. Normal indexed LA volume has been determined to be 22 ± 6 cc/m^2^ using the preferred biplane techniques (area-length or method of disks) in a number of studies involving several hundred patients.

Tsang et al. [11] data suggest that echocardiography in general, and evaluation of LA volume in particular, should be included among tests and variables offering insight into cardiovascular risk. In fact, the authors demonstrated that LA volume is a more robust cardiovascular risk marker than LA area or diameter in patients who are in sinus rhythm [21] and a larger LA volume was associated with a higher risk of AF in older patients [20, 22].

Consequently, indexed LA volume measurements should become a routine laboratory measure because they reflect the burden and chronicity of elevated LV filling pressure and are a strong predictor of outcome [16].

### 3.3. Pulsed Doppler: Transmitral Flow and Pulmonary Venous Flow

**Figure 4:**
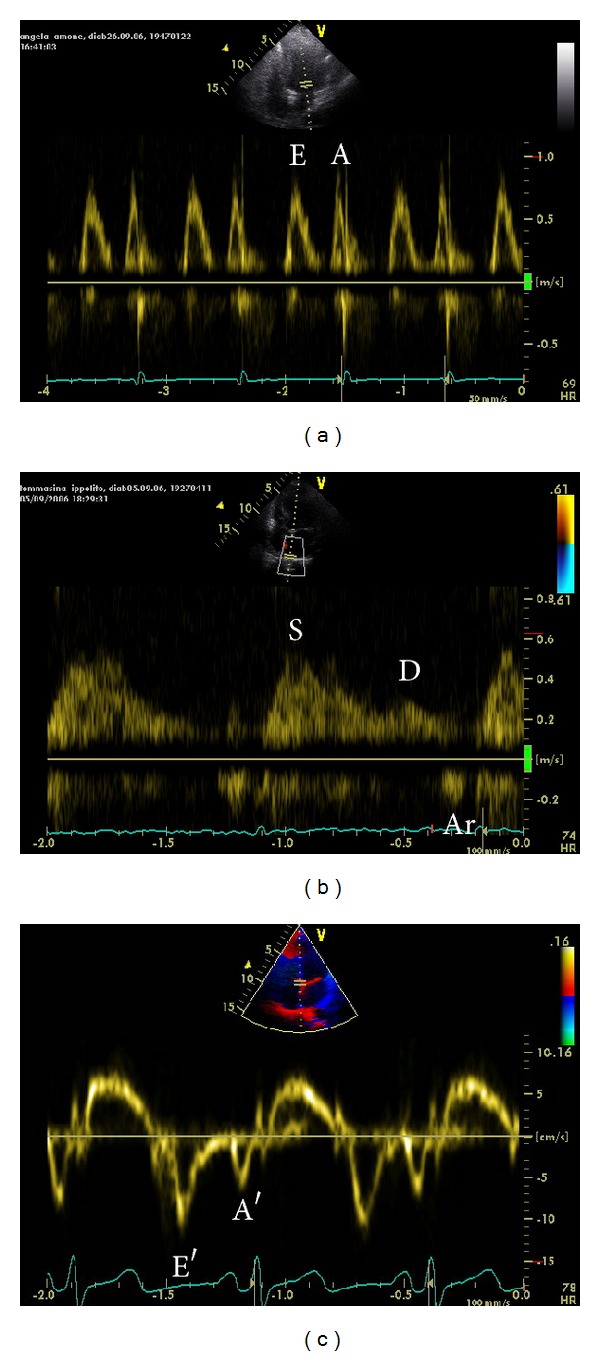
Atrial contraction: wave (a) at mitral inflow (a), peak retrograde diastolic (Ar) velocity at pulmonary venous flow (b) by pulsed Doppler, and late diastolic (A′) velocity by pulsed wave tissue imaging (c).

Mitral inflow patterns by pulsed wave Doppler examination demonstrate passive ventricular filling in early diastole (E wave) and late active filling phase during atrial contraction (A wave) (Figure 4(a)). The sample volume is placed at the tips of the mitral leaflets by the apical four-chamber view. Estimation of the peak A wave velocity is commonly employed in studies that have evaluated atrial function [23, 24].

The peak A wave velocity is influenced by heart rate, loading conditions, and age and depends on LA systolic function, LV compliance, and AV conduction [25].

LA systolic function depends on LA contraction and LA pressure during the onset of atrial systole. With aging and during the first degree of LV diastolic dysfunction, LV filling during early diastole decreases, LA pressure is still normal, and LA contracts to compensate the anomalous LV filling and to maintain an adequate LV EF. With the progression of LV dysfunction, LA pressure increases, and the flow due to atrial contraction decreases. LV filling during early diastole begins earlier and lasts for a short time. LA pump function vanishes because it works against elevated LV late diastolic pressure and a portion of blood, pumped from LA, flows behind in the pulmonary veins. For this reason, peak A wave velocity is reduced.

The peak A wave velocity has also been employed in the serial follow-up of patients with AF following the restoration of SR by either cardioversion [23] or catheter ablation [26].

The A wave is absent in the presence of AF, and restoration of sinus rhythm results in its reappearance. The temporal recovery of the A wave velocity is largely dependent on the duration of AF prior to cardioversion. With brief duration of AF (2 days to under 2 weeks), the peak A wave velocity is similar to that of the general population following the restoration of sinus rhythm [23, 27].

However, in cases with intermediate duration (2–6 weeks) or prolonged AF (over 6 weeks), the peak A wave velocity was significantly lower than that in a normal control cohort despite the restoration and maintenance of SR [23]. Velocities normalise within 1 week in the intermediate duration group and after 4 weeks in the group with prolonged duration of AF. Thus, it was postulated that “atrial stunning” occurring after the restoration of sinus rhythm is reversed over a period of 3-4 weeks.

By pulsed Doppler we can also measure pulmonary venous flow. The sample volume is placed into pulmonary veins (commonly right upper vein) by the apical four-chamber view. We can measure (1) peak systolic (S) velocity; (2) peak anterograde diastolic (D) velocity; (3) peak retrograde diastolic (Ar) velocity; and (4) its duration during LA contraction (Figure 4(b)). Peak systolic (S) velocity is biphasic: S1, during diastole of LA and S2 during LA filling, when mitral valve is closed. S1 represents LA relaxation and S2 LA reservoir function. With the deterioration of LV diastolic function, in first degree diastolic dysfunction, LA reservoir function increases (peak systolic velocity), together with LA pump function (peak retrograde diastolic velocity), while LA conduit function (peak anterograde diastolic velocity) decreases. In the second degree diastolic dysfunction, LA conduit function increases, LA reservoir function decreases, and pump function significantly increases. In the restrictive pattern, LA conduit plays a dominant role; LA reservoir and pump functions are severely depressed in this stage, that correspond to severe LV dysfunction [28].

### 3.4. Doppler Tissue Imaging

Pulsed wave tissue Doppler imaging (DTI) is performed in the apical views to acquire mitral annular velocities. Measurements include the systolic (S), early diastolic (E′), and late diastolic (A′) velocities (Figure 4(c)). Studies have demonstrated that the peak velocity in late diastole secondary to atrial contraction (A′) measured using pulsed wave DTI is a rapid and an accurate marker of atrial function [29, 30]. The pulsed wave DTI sample volume is placed on the mitral annulus in the apical four- and two-chamber views. The A′ velocity was seen to increase with aging, similar to the peak A wave velocity [29]. The A′ velocity correlates with parameters of atrial function. Hesse et al. [30] demonstrated that the A′ velocity correlated with LA fractional area and volume change. The A′ velocity is reduced in diseased states associated with LA dysfunction.

## 4. LA Function and Left Atrial Myocardial Deformation Properties Assessed by New Echocardiographic Techniques

LA function contributes to LV filling by means of its three components: reservoir, conduit, and pump. The reservoir phase begins with ventricular systole. During this phase, which corresponds to LV isovolumetric contraction, ejection, and isovolumetric relaxation, LA receives blood from the pulmonary vein, while mitral valve is closed, LA fills and enlarges, and LA volume increases. During early diastole, LA distends for passive emptying, atrial blood is suctioned by the ventricle and the atrium acts as a passive conduit, and LA volume decreases. During late diastole, the atrial muscle contracts actively and performs a pump function that completes ventricular filling. In normal subjects, the atrial contribution as a reservoir is about 40%, as passive conduit is about 35%, and as a pump is approximately 25% [16]. The reservoir function of LA is particularly relevant because 40% of the systolic volume is stored in the atrium during LV systole.

Traditionally, assessment of LA function has been performed by measuring morphological and static parameters by 2D echocardiography, such as LA diameter, area, and volume. Recently, alternative methods have been developed to measure myocardial deformation properties both by Doppler myocardial imaging-derived strain and by speckle tracking echocardiography strain, to detect early functional remodelling before anatomical alterations occur [2, 4–6, 31–34]. This LA remodelling process, for decreasing of its elastic properties, and LA enlargement, because of its thin-walled structure, have been regarded as important prognostic factors in various heart diseases. Many studies confirm that LA dilation is associated with poorer prognosis and highlight LA deformation analysis as a more sensitive parameter providing independent and additional prognostic information to other conventional LA measurements [4–6, 35–43].

Atrial myocardial deformation properties are expressed by a dimensionless parameter, the strain (S) that is defined as the percentage change from the original dimension, and the strain rate (SR) that is the rate of myocardial deformation and measures the velocity with which myocardial deformation occurs and is expressed as S^−1^. Atrial S and SR are studied only in the longitudinal plane; it is not possible to obtain atrial radial S by the parasternal view because the atrial wall is too much thin to perform the analysis. During atrial contraction and relaxation, a deformation gradient is observed from all views, with higher S in atrioventricular junction and lower S in the atrial roof, contrarily to LV; this could be because the atrial roof is fixed to the mediastinum by the pulmonary veins [35].

### 4.1. Strain Doppler Method

For S Doppler evaluation, real-time 2D colour Doppler myocardial imaging data are recorded from the LA, using standard apical views at a high frame rate (>180 fps). The data are stored in digital format and analysed offline by dedicated software, that allows calculating three parameters: local peak systolic velocity (V), local peak systolic SR and its integral, and local peak systolic S [4, 5].

**Figure 5:**
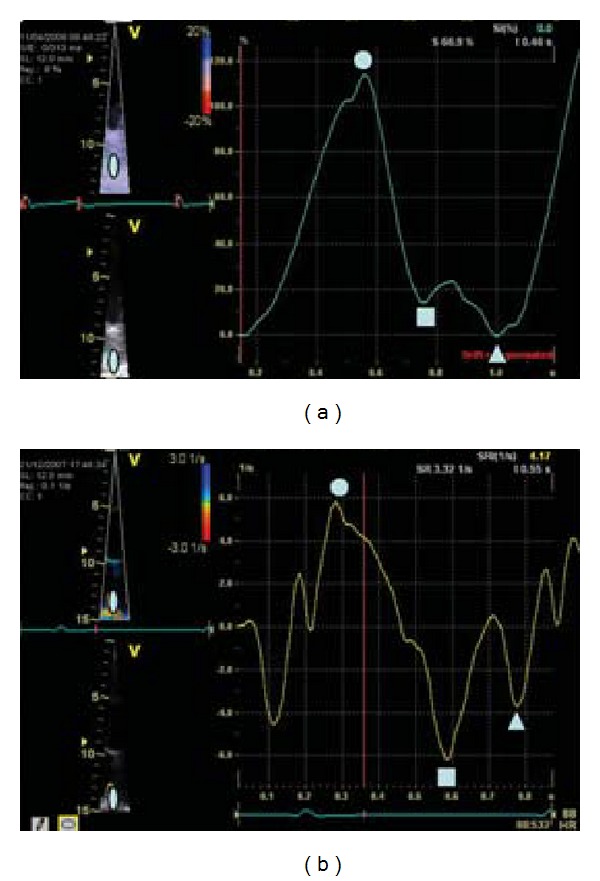
Left atrial strain (a) and strain rate (b) curves by Doppler. Left atrial reservoir function is studied by peak systolic value (ball), left atrial conduit function by peak early diastolic value (square), and left atrial pump function by peak late diastolic value (triangle).

During LV systole, LA acts as a reservoir: passive stretching of the LA walls, during LV systole, leads to LA longitudinal lengthening, and lengthening is recorded as positive S and SR values [44]. During LV diastole, there is atrial shortening that is recorded as negative S and SR values. Analysis may be performed for atrial longitudinal V, SR, and S at 3 segments (basal, medium, and near the atrial roof) from the apical views of the LA septum, LA lateral wall (from the apical 4-chamber view), and LA inferior and LA anterior wall (from the apical 2-chamber view). To derive V, SR, and S profiles from the studied segment, a 6 × 3 mm region of interest with elliptical shape is chosen and is continuously manually tracked frame by frame to maintain its position within atrial wall with a proprietary semiautomated tracking algorithm. Peak positive systolic, early diastolic, and late diastolic values were calculated from the extracted curves (Figure 5) and averaged, in some studies, as the sum of the peak values recorded in each LA wall divided by the number of the studied walls. While the study of atrial myocardial velocity shows curves similar to those of the ventricular myocardium, confirming the influence of the drag on the myocardial velocities, atrial S shows curves with morphology opposite to those of ventricular ones. In fact during cardiac cycle, ventricles and atria move in opposite directions. During LV systole, atrial myocardium stretches along the longitudinal plane and LA wall longitudinal lengthening was represented with a peak positive S value. Instead ventricular myocardium shortens during LV systole and myocardial longitudinal shortening was represented with a peak negative S value, because of the A-V plane displacement toward the apex. Ventricular SR curves are also opposite to atrial SR curves.

Atrial myocardial deformation properties values by S Doppler range, in different studies, for LA systolic S, from 65.4 ± 19.5% to 82 ± 19% and for LA systolic SR from 3.4 ± 1 S^−1^ to 4.4 ± 1.6 S^−1^ [2, 4, 31].

### 4.2. Speckle Tracking Method

More recently, a new method, 2D S and SR imaging (speckle tracking), has been developed to study ventricular function and has been used to evaluate atrial function [1, 6, 31–35, 45–49]. It is independent of both cardiac translation and angle dependency; it is based on an automated tracking system, with very good reproducibility, unlike Doppler-based S imaging. 2D S and SR imaging provides automatic calculation of mean values from the pattern of speckles in predefined myocardial segments, reflecting segmental function better than the analysis of only one point in space used in previous Doppler S analysis. The important advantage is that 2D S uses 2D loops (grayscale images) from the routine echocardiographic examination, and it is becoming a widespread technique, with many clinical implications. Although specific software for LA 2D S-SR has not yet been provided, all studies that analyzed LA function applied a program intended for LV. All studies showed that the feasibility and the reproducibility of LA patterns and measurements were good [6, 32–35, 45–49].

2D S uses grayscale (B-Mode) sector image and is based on frame by frame tracking of small rectangular image blocks with stable speckle pattern [50, 51]. Frame rates of 50 to 90 Hz are used for routine grayscale imaging. Apical 4-chamber view was obtained using the same ultrasound system and the probe used for standard echocardiography. By tracing the endocardial contour on an end-systolic cavitary frame after defining the thickness of the region to be considered, the software will automatically track the atrial wall on subsequent frames. Adequate tracking can be real-time verified and corrected by adjusting the region of interest or the contour (increased or decreased the width for thicker or thinner walls, resp.). Using this technique, analysis is performed from the 4-chamber and 2-chamber apical views for the segments of LA septum, LA lateral wall, LA inferior wall, and LA anterior wall and then are averaged [4, 5].

As for S Doppler, for 2D speckle tracking too, during LV systole, LA wall longitudinal lengthening is represented with a peak positive S value; instead, myocardial longitudinal shortening is represented with a peak negative S value.

**Figure 6:**
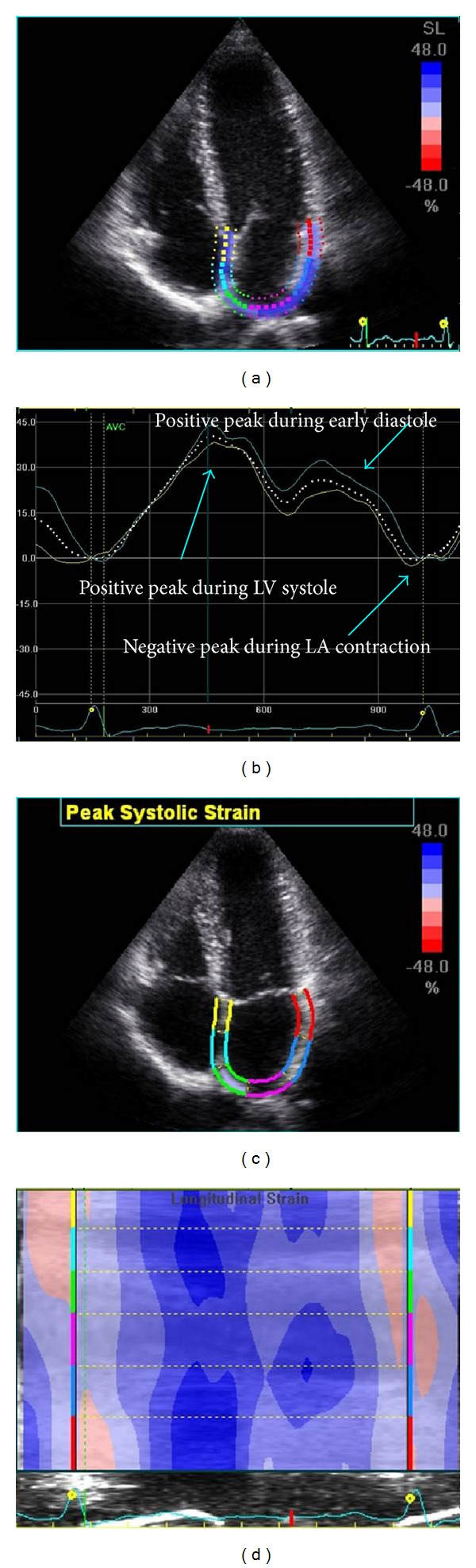
Left atrial deformation (strain) by speckle tracking. During reservoir function, atrial S increases, reaching a positive peak (systolic peak), at the end of atrial filling, before the mitral valve opening, during LV systole. After mitral valve opening, during conduit phase, atrial S decreases, until reaching a plateau, during diastasis, followed by a second positive peak, during early diastole, before LA contraction. Then there is a negative peak, at the end of atrial contraction.

During reservoir function, because of LA filling and lengthening, atrial S increases, reaching a positive peak, at the end of atrial filling, before the mitral valve opening, because of the downward displacement of the mitral annulus toward the apex, due to LV contraction (Figure 6). After mitral valve opening, because of LA rapid emptying and shortening (conduit phase), atrial S decreases, until reaching a plateau, during diastasis, followed by a second positive peak, but lower than the first, that corresponds to the period that precedes atrial contraction. Then there is a negative peak, at the end of atrial contraction. The second positive peak, during atrial contraction (pump function), is present only in sinus rhythm people.

Atrial myocardial deformation properties values by 2D speckle tracking range, in different studies, for LA systolic S, from 35.7 ± 5.8% to 42.2 ± 6.1% and for LA systolic SR from 1.43 ± 0.24 S^−1^ to 2.47 ± 0.55S^−1^ [6, 32, 35].

### 4.3. Limitations of the Strain 

Like other Doppler modalities, DTI-derived strain measurements are dependent on the direction of the Doppler angle of incidence in relation to myocardial motion. The need to manually track the LA wall and reposition the region of interest on each of the walls, frame by frame, makes using this method in a clinical setting prohibitively time consuming (about 20 minutes per patient) and decreases the reproducibility. A technical limitation of speckle tracking is that this technique is load dependent, and it is dependent on frame rate and image resolution. Because specific software to LA both by strain Doppler and by speckle tracking has not yet been provided, we applied a program intended for left ventricular strain to analyse left atrial strain. In future studies, changes in the software may be needed to improve the tracking ability of the Doppler and speckle tracking system for LA functional study.

## 5. Clinical Applications

Several studies have analysed S Doppler and speckle tracking in different physiopathology conditions associated with atrial dysfunction, such as atrial fibrillation (AF), valvular diseases, heart failure, hypertension, diabetes, and cardiomyopathies [2–6, 31–35, 45–51]. Population-based studies have demonstrated the prognostic value of the LA analysis for long-term outcome [6, 36]. Global LA S, both Doppler and speckle tracking, is a strong and independent predictor of cardiovascular events (AF, congestive heart failure, stroke, transient ischemic attack, myocardial infarction, coronary revascularization, and cardiovascular death) and appears to be superior to conventional echocardiographic parameters of LA analysis (LA dimension, LA area, LA volume, and LA ejection fraction) [4, 6, 36] (Table 1).

### 5.1. Atrial Fibrillation

AF has increasingly become a focus of attention because it remains the most encountered arrhythmia in clinical practice, associated with a poorer prognosis and increased risk of mortality, stroke, cardioembolic events, hospitalization, heart failure, and decreased life quality [52].

Atrial structural, electrical, and functional changes caused by AF are the substrates responsible for arrhythmia's perpetuation and tendency to recur, resulting in poor quality of life.

AF is characterized by LA remodelling and dilation, due to myocyte cell loss, by changes in extracellular matrix composition and fibroblasts proliferation and differentiation into myofibroblasts, with both diffuse interstitial and patchy fibrosis [53]. Fibrosis usually results from an accumulation of fibrillar collagen deposits, occurring most commonly as a reparative process to replace degenerating myocardial tissue with concomitant reactive fibrosis, which causes interstitial expansion. Structural remodelling results in electrical tissue inhomogeneity and slowed conduction and electrical uncoupling, facilitating AF continuation without inducing changes in atrial action potential properties [53].

In AF patients a tool capable of assessing atrial function is very useful; in fact the challenge for cardiologists is to detect early functional remodelling before anatomical alterations occur. In patients with AF, reservoir and conduit atrial S and SR are decreased and atrial S and SR are absent during late diastole (missing booster pump function) because electrical activation pathway is disrupted and atrial mechanical performance becomes abnormal. The impairment of reservoir function can be detected even before atrial dilation has occurred, due to atrial fibrosis and reduced atrial compliance. With restoration of sinus rhythm there is an increase in atrial reservoir and passive conduit S, reflecting reverse atrial remodelling [54]. Instead, while the peak velocity of the pulse tissue Doppler A wave increases early after sinus rhythm is restored, left atrial peak SR during late diastole (which reflects atrial pump function) is not normalized until a 6-month period after cardioversion, because of the atrial stunning [37]. Atrial stunning is characterized by a reduction in the mechanical function of the LA in AF after restoring sinus rhythm; it may last for several weeks and is associated with an increase in thromboembolic risk for the duration of the vulnerable period.

Severity of the impairment in atrial function, assessed by the decrease in 2D S and SR during the reservoir phase and early diastole, is an independent predictor of AF recurrence. And, then, also in patients treated by ablation for a lone paroxysmal AF, LA Doppler S and SR values significantly improved after 1 year, however, remaining inferior to controls, reflecting a persistent or irreversible alteration of LA compliance [55]. Numerous studies have demonstrated that atrial S, both by Doppler and by speckle tracking, can predict AF recurrence in patients treated by electrical cardioversion or ablation [38, 39].

Schneider et al. [39] found cut-off values of >2.25 S^−1^ for LA SR, by Doppler, and of >19.5% for LA S as best predictors of sinus rhythm maintenance after catheter ablation.

Also in our study [5], 65 patients with “lone” AF, with recent-onset (≤3 months) AF and with almost normal LA dimension (<4.5 cm), lower S (22%), and SR (1.8 S^−1^) values, evaluated by S Doppler, show larger atrial stiffness (fibrosis), due to atrial remodeling because of AF and so a higher likelihood of AF recurrence. By multivariable analysis, at 9-month follow-up, LA S and SR peak systolic values were individual independent predictors of sinus rhythm maintenance, after external cardioversion.

Similar results were obtained by Mirza et al. [40], who demonstrated that regional LA lateral wall S was a preprocedural determinant of AF recurrence in patients who underwent cardioversion, independent of LA enlargement.

Recent study showed that lower longitudinal AS values were able to predict higher cardioembolic risk (CHADS2 score ≥ 2) in 34 patients with nonvalvular AF [41].

Recently, Kuppahally et al. [56] showed that LA wall fibrosis, evaluated by delayed-enhancement MRI, is inversely related to LA S and SR, and these are related to the AF burden. Patients with persistent AF as compared with paroxysmal AF had more fibrosis and lower midseptal and midlateral S, evaluated by speckle tracking, emphasizing the progressive remodeling process that occurs once AF is initiated. Neither the extent of fibrosis nor the degree of reduction in S was influenced by age, sex, severity of mitral regurgitation, or history of hypertension, suggesting that the change may be primarily due to AF. These results explain why LA S and SR, measured during reservoir function, are useful in predicting the patients who will develop AF or the recurrence of AF, after ablation [57]. These findings definitively correlate the histopathological report to fibrosis with loss of function and clarify the relationship between atrial remodeling and functional alterations in patients with AF. In the clinical management of patients with AF, given the close relationship between morphology and function, a reduced atrial deformation during the reservoir phase of cardiac cycle may be an early and noninvasive marker of the amount of atrial wall fibrosis [58].

### 5.2. Valvular Heart Disease

Also in valvular heart diseases, LA S and SR are predictors of adverse events, showing abnormal atrial myocardial deformation properties [4, 6].

We studied 53 asymptomatic patients with mitral stenosis (MS) compared to 53 healthy controls both by the standard echo-Doppler study (mitral valve area, mean gradient, systolic pulmonary pressure, LA width, LA volumes, and LA compliance index) and by S-SR Doppler. LA myocardial deformation indices were significantly compromised in MS patients. At 3-year follow-up, 22 (41%) patients had events (symptoms, hospitalization for cardiac cause, atrial fibrillation, thromboembolic events, valvular surgery, or percutaneous commissurotomy). Comparing the MS patients who had events during the 3-year follow-up with those who did not, the former were older, with larger LA width, and had bigger LA volumes, although these parameters did not reach a significant value, whereas atrial myocardial systolic SR was significantly impaired in patients with events. In multivariate analysis, the best predictor of events at 3-year follow-up was the LA peak systolic SR average, with a cut-off value of 1.69 S^−1^, associated with a sensitivity of 88% and a specificity of 80.6%, demonstrating that peak systolic SR is a more sensible index of atrial dysfunction than conventional parameters (atrial diameter, volume, and LA compliance index) because in atrial diseases it is changed before a clear increase in atrial dimensions and volumes. The changes in peak systolic atrial myocardial deformation properties in patients with MS may be due to disorganization of the atrial muscle bundles and atrial fibrosis. In the presence of the same degree of MS as assessed by the standard echocardiographic study, patients may become symptomatic and show a different prognosis, because of different degrees of atrial muscle bundles disorganization and atrial fibrosis, causing atrial stiffness and atrial reservoir dysfunction [4].

These data were also recently confirmed in another study done by us, in which we have demonstrated that LA deformation properties, assessed by speckle tracking, are abnormal in 101 asymptomatic patients with mild to moderate MS. At 4-year follow-up, 20 patients (20%) showed AF on standard electrocardiography or 24-hour Holter electrocardiography. Patients with MS who had AF were older than those who did not, without significant differences in LA dimensions, volumes, ejection fraction, and compliance index. Instead, atrial myocardial systolic 2D S was significantly impaired in patients with events and was able to predict AF at 4-year follow-up. On multivariate analysis (age, LA volume, planimetric mitral area, average annular E′, and LA strain) the best predictor of AF was average LA peak systolic S, with a cut-off value of 17.4%. Patients with asymptomatic MS with mean atrial S > 17.4% showed increased survival time free of AF compared with those with mean atrial S ≤ 17.4%. Our findings suggest this cut-off as a predictor of AF, showing the ability of 2D S to detect abnormalities in atrial compliance earlier than conventional echocardiographic parameters, reflecting structural changes, and to follow up electrical and structural remodelling. 2D S can assess early atrial reservoir dysfunction in patients with MS, allowing the early detection of fibrosis and may help us to recognize patients with asymptomatic MS who will develop AF and so with worse prognosis, earlier than conventional echocardiographic parameters [6].

In patients with different degree of mitral regurgitation (MR), LA S decreases with the increase of severity of mitral regurgitation and LA S shows lower values in patients with history of paroxysmal AF [59]. In patients with severe MR referred for cardiac surgery, impairment of LA longitudinal deformation, as assessed by the global peak atrial S, correlated strongly with the extent of LA fibrosis and remodelling, evaluated by histopathologic examination [60].

In aortic stenosis, LA enlargement and dysfunction adversely affect outcomes. Some investigators [42, 43] studied the impact of aortic stenosis on LA phasic function and reported that all LA longitudinal S values were reduced and that LA booster pump function was particularly affected by the severity of aortic stenosis.

### 5.3. Heart Failure

The evaluation of LA function is important also in heart failure (HF). In fact, LA function seems to be influenced not only by atrial stiffness but also by LV compliance during LV filling and by LV contraction through the displacement at the bottom of the base during LV systole [61].

It has been demonstrated that LA stiffness index, calculated as E/E′ ratio/global LA S (%), as described by Kurt et al. [62], is an accurate marker for differentiating diastolic dysfunction patients from patients with diastolic HF (cut-off of 0.99, with a sensitivity of 85%, and a specificity of 78%). Also a reduction of LA SR Doppler, unlike LV mass and LA volume, is able to discriminate patients affected by diastolic HF from simple diastolic dysfunction [62]. Khan et al. have studied 50 patients with diastolic dysfunction (first and second degree) and 100 normal controls. LA S, by speckle tracking, is significantly reduced in early diastolic dysfunction and LA stiffness index is significantly higher among patients with diastolic dysfunction compared with controls, indicating reduction in LA compliance during the reservoir phase and an increase in the filling pressures in diastolic dysfunction [63].

An inverse relationship between both reservoir and conduit functions and Doppler parameters of LV diastolic dysfunction [64] and LV end-diastolic pressure has been demonstrated in patients with HF [65].

LA pump function presents a biphasic response: in early HF it is augmented as compensation for low early LV filling, whereas in late stages, as work mismatch progresses, LA contractile properties gradually deteriorate [2]. LA longitudinal S and stiffness are the most accurate indexes of diastolic HF and correlate with worse New York Heart Association functional class [66]. These data suggest that the decrease in LA compliance expressed by a reduction in reservoir function might occur before structural remodelling, allowing identification of LV diastolic dysfunction in subjects with preserved ejection fraction earlier than overt structural changes.

Although LA volumes increased linearly with the severity of diastolic dysfunction, LA reservoir and conduit function progressively declined at the advanced stages of diastolic dysfunction. This is associated with an initial augmentation of LA booster function in mild diastolic dysfunction to maintain total LA emptying volumes [67]. However, in patients with severe diastolic dysfunction, LA booster function declined and is significantly reduced compared with subjects with normal diastolic function and those with mild or moderate diastolic dysfunction. This reflects that the LA Frank-Starling physiologic mechanism, verified in a 3D echocardiographic study [68], no longer operates during the advanced stages of diastolic dysfunction.

In case of diastolic dysfunction, changes in ventricular filling occur, and the relative contributions of each of these components vary in order to maintain systolic ventricular volume. Prolonged ventricular relaxation (first degree of diastolic dysfunction) leads to a decrease in conduit function, while the reservoir and pump functions increase. As diastolic dysfunction progresses and the patients exhibit a pseudonormal (second degree) or restrictive (third degree) mitral flow, the passive conduit function increases, while the reservoir and active pump functions decrease significantly; in fact most of ventricular filling occurs during early diastole and not during late diastole because of the increased left ventricular filling pressure and because of the decreased force of contraction.

LA global longitudinal S demonstrated the highest diagnostic accuracy and excellent sensitivity and sensibility to predict elevated filling pressure (>18 mmHg), compared to E/E' ratio. LA global S, parameter for the functional evaluation of the atrial reservoir function, which resulted progressively decreased with the augmentation of LV filling pressure [69, 70]. The potential mechanism of this inverse correlation could be explained by the principle that pulmonary capillary wedge pressure is the afterload of LA function; if this pressure is high, LA should be chronically stressed, resulting in decrease of LA reservoir function and finally in remodelling with LA chamber dilation, as demonstrated in patients with HF.

### 5.4. Cardiomyopathy

2D strain represents a promising noninvasive technique to assess LA myocardial function also in patients with dilated cardiomyopathy (DCM). LA pump and reservoir functions at baseline and after cardiac resynchronization therapy are more depressed in idiopathic compared with ischaemic DCM patients. A significant improvement in LA systolic function was obtained only in patients with ischaemic DCM, responders to cardiac resynchronization therapy. In fact, in idiopathic DCM, although loading conditions are the same, a more depressed LA booster pump function at rest has been observed compared with ischaemic patients and attributed both to alterated LA overload and to LA larger involvement in the myopathic process [51].

Our preliminary data by speckle tracking showed in 30 diabetic patients without coronary artery disease abnormalities of atrial reservoir function, that is, expression of early pathological changes of the atrial walls, thinner than ventricular walls, when LV global and segmental systolic function is still normal [71].

Then in 50 diabetic patients with coronary artery disease, LA S and SR, evaluated by Doppler imaging, identify elevated pulmonary wedge pressure and diastolic dysfunction grade [72].

LA longitudinal S during ventricular systole, early diastole, and late diastole was lower in patients with diabetes and in patients with hypertension than in controls and further reduced in patients with coexisting diabetes and hypertension. The association of diabetes and hypertension with LA S abnormalities is independent of clinical and echocardiographic variables (LA dimension, volume, and LA ejection fraction), that were similar [73].

Atrial S has an important prognostic value also in patients suffering from hypertrophic cardiomyopathy (HCM). A cut-off value of SR, by speckle tracking, during late diastole of 0.92 S^−1^ is able to predict the onset of symptoms of HF [74] and cut-off value of LA S during systole < 21% is able to predict the onset of AF within the next 12 months. Paraskevaidis et al. [75] showed that LA longitudinal S was reduced in HCM patients compared to patients with non-HCM left ventricular hypertrophy (LVH) or healthy subjects. This finding was evident in all three atrial phases and in the overall longitudinal atrial function and was observed both by tissue Doppler and by 2D atrial S imaging. In ROC analysis, 2D atrial contractile S discriminated HCM from non-HCM LVH with a cut-off of −10.82%, a sensitivity of 82%, and a specificity of 81%. So 2D S seemed to have an additive prognostic value in differentiating HCM from non-HCM LVH, when combined with conventional echocardiographic indices, and is more reproducible and less time consuming than tissue Doppler S.

LA S also plays a key role in the early differential diagnosis between HCM and physiologic LV hypertrophy. Although LV mass index, LA volume index, and ejection fraction were comparable between patients with HCM and athletes, patients with HCM had a significantly lower systolic LA S (19 + 8% versus 43 + 8%), systolic LA SR (0.7 + 0.2 s^−1^ versus 1.6 + 0.2 s^−1^), and late diastolic LA SR (−0.8 + 0.1 s^−1^ versus −1.4 + 0.3 s^−1^) compared to athletes. Among hypertrophic subjects, independent predictors of hypertrophy related to HCM were LA systolic S and E/E′ ratio [76].

Also in amyloidosis, studies have demonstrated that LA systolic dysfunction appears to be independent of global LV systolic and diastolic function and LA dilatation. Abnormal LA function, using S echocardiography criteria, was identified in a significant number of patients with echocardiographic evidence of cardiac involvement and mean peak systolic LA SR was lower in those with, versus those without, CHF. Furthermore, mean peak systolic LA SR was lower in patients without echocardiographic evidence of cardiac involvement compared with controls, suggesting LA involvement in the absence of the classic echocardiographic features of cardiac amyloidosis [77].

### 5.5. Hypertension

Atrial function evaluation is also useful to distinguish the pathological from physiological hypertrophy. During ventricular diastole LA is exposed to LV filling pressure. In normal subjects, during exercise, LA S increases during reservoir and pump function, to allow an optimal ventricular filling during the haemodynamic changes. In patients who have hypertrophy secondary to hypertension, atrial pressure increases to allow adequate LV filling and an increase in wall tension contributes to its expansion. As a consequence, pump function increases while reservoir function decreases, determining an increase of LA S during atrial contraction and its decrease during reservoir function [76, 78].

In 2007, Kokubu et al. [79] reported that LA S and SR values were lower in patients with hypertension when compared with normal subjects, irrespective of the presence of LA enlargement or LV hypertrophy. Moreover, deformation parameters tended to normalize after renin–angiotensin system inhibition, indicating a therapeutic effect on LA function. The pathophysiology of LA dysfunction in a hypertensive heart is attributed to elevated pressure to which the LA is chronically exposed during ventricular diastole, leading to a rise in LA pressure and a reduction in reservoir and conduit functions [80]. In early hypertensive heart disease, LA stretching causes a temporary enhancement of LA pump function, which is necessary to maintain adequate ventricular filling. When compliance is lost and stiffness increases, eventually LA contractility suffers. According to the stage of the disease and the entity of organ damage, LA mechanics in hypertension can be depressed in all the three phases or characterized by a temporary augmentation of LA pump performance, especially during the earlier phases of the disease. When compared with normal age-matched controls and to a group of subjects with physiological hypertrophy, hypertensive patients experience reductions in S and SR values in all three phases of atrial function, proportional to their exercise capacity.

### 5.6. Atrial Septal Defect Closure

In 20 patients, in sinus rhythm, one year after successful percutaneous atrial septal defect closure, using the Amplatzer occlude, LA 2D S and SR were able to evaluate atrial regional function. Analysing the Amplatzer ASD occluder, a bulky noncontractile element, passively moved by global heart motion, 2D S demonstrated almost the absence of any deformation. Conversely, on the normal lateral atrial wall a significant higher deformation was detectable. 2D S has the ability in discriminating the normal atrial deformation from the passive movement of an interatrial device, demonstrating that 2D S is not influenced by global heart motion and tethering from adjacent segments [81].

### 5.7. Three-Dimensional (3D) Echocardiography and Volumes

Keller et al. [82] have demonstrated that freehand 3D echocardiography is a valid, accurate, reproducible method for determining left and right atrial volumes in humans and it is comparable to magnetic resonance imaging and is superior to current mono and 2D echocardiographic techniques. 3D echocardiography has the highest correlation, lowest bias and limits of agreement, and lowest interobserver variability of the echocardiographic methods evaluated using MRI as a reference standard, due to eliminating the use of geometric assumptions, reducing or eliminating image positioning errors, and significantly increasing sampling of the atrial boundaries.

Transthoracic real-time 3D echocardiographic images of the LA are acquired, in the left lateral decubitus, in end expiration to avoid translational motion using 4V probe. A full-volume single-beat acquisition is obtained in apical view. Subvolumes are acquired to generate the full-volume 3D data set of the LA from the apical approach, taking care to encompass the entire LA cavity in the data set. The pyramidal volume data are displayed in three different cross-sections that can be modified interactively by manual shifting of vertical and horizontal lines in the two orthogonal apical and the short-axis views. Focus is laid on the most optimal imaging of LA in the 4-chamber view. Data sets are stored digitally and exported to a separate workstation for offline analysis.

To assess LA volumes by 3D echocardiography, we may use two methods: one by LVQ Auto 4D and one by TomTec 4D.

**Figure 7:**
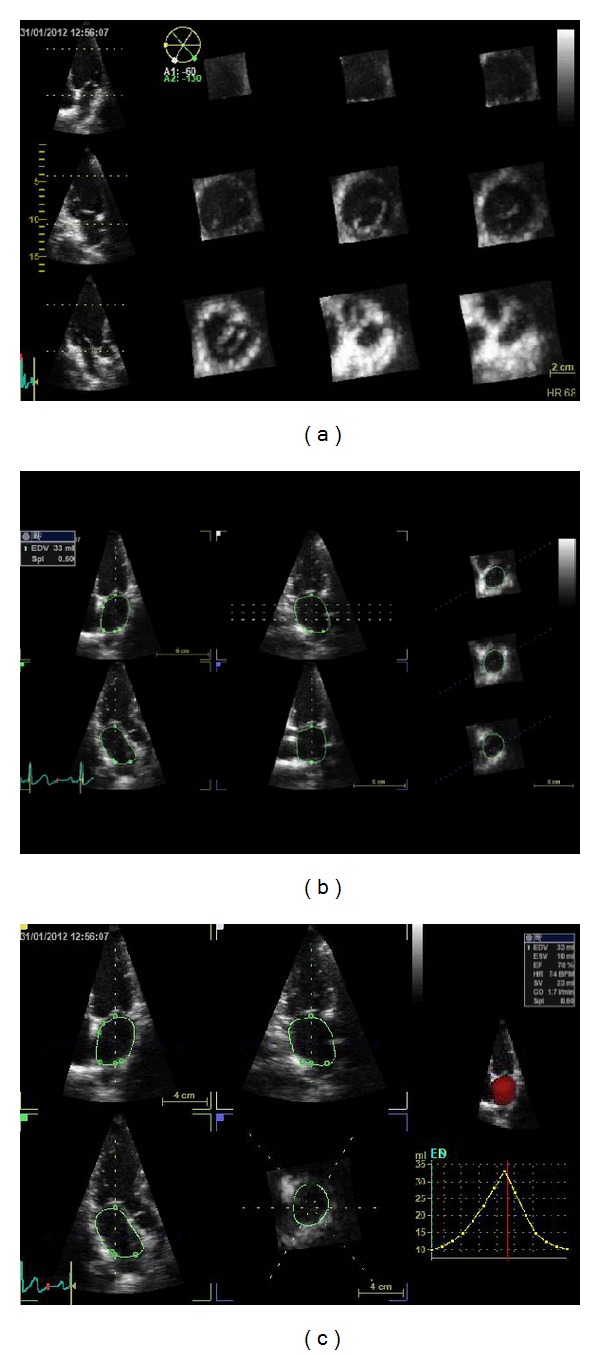
Left atrial volumes by LVQ Auto 4D echocardiography. (a) Left atrial full-volume data sets are acquired. (b) After manually initializing one point to identify the mitral valve plane and another point for the centre of LA roof, the program automatically identifies the endocardial surface both in end-systole and in end-diastole. (c) Atrial maximum (max) and minimum (min) volumes are obtained and displayed in curve, in numeric values, and in 3D image (in red).

For LVQ Auto 4D method, since the first frame in the loop corresponded to ventricular end-diastole, initial measurements are performed in the frame with the largest atrial dimension, corresponding to ventricular end-systole, just before the opening of the mitral valve, manually adjusting the frame. Two anatomic landmarks are manually initialized for LA: one point is set to identify the mitral valve plane and one point to identify the centre of LA roof in the apical view. Following this manual of identification, the program automatically identifies the endocardial surface. Manual adjustments of the endocardial surface are performed, in order to include trabeculae and to exclude atrial appendages and large veins from the cavity volumes [8] (Figure 7).

Afterwards, the frame with the smallest atrial dimension is selected with similar surface detection and manual editing. Atrial maximum (max) and minimum (min) volumes are obtained. Usually, the time consumed to obtain these measurements is about 2-3 minutes. Aune et al. [8] have provided normal ranges for atrial volumes and EF with RT3DE by LVQ Auto 4D method from a comprehensive series of 160 normal individuals aged 30–80 years. Upper normal values for LA volumes and EF were similar for both genders. Upper normal reference values were 41 mL/m2 for maximum (max) LAVI and 19 mL/m2 for minimum (min) LAVI. The lower normal reference value is 45% for LAEF. They concluded that the new RTDE technique is fast, simple, fairly accurate, and reproducible, allows bedside measurements of atrial volumes and contractility without offline analysis, and represents a more reproducible and robust method for atrial volume measurements than 2DE [8].

**Figure 8:**
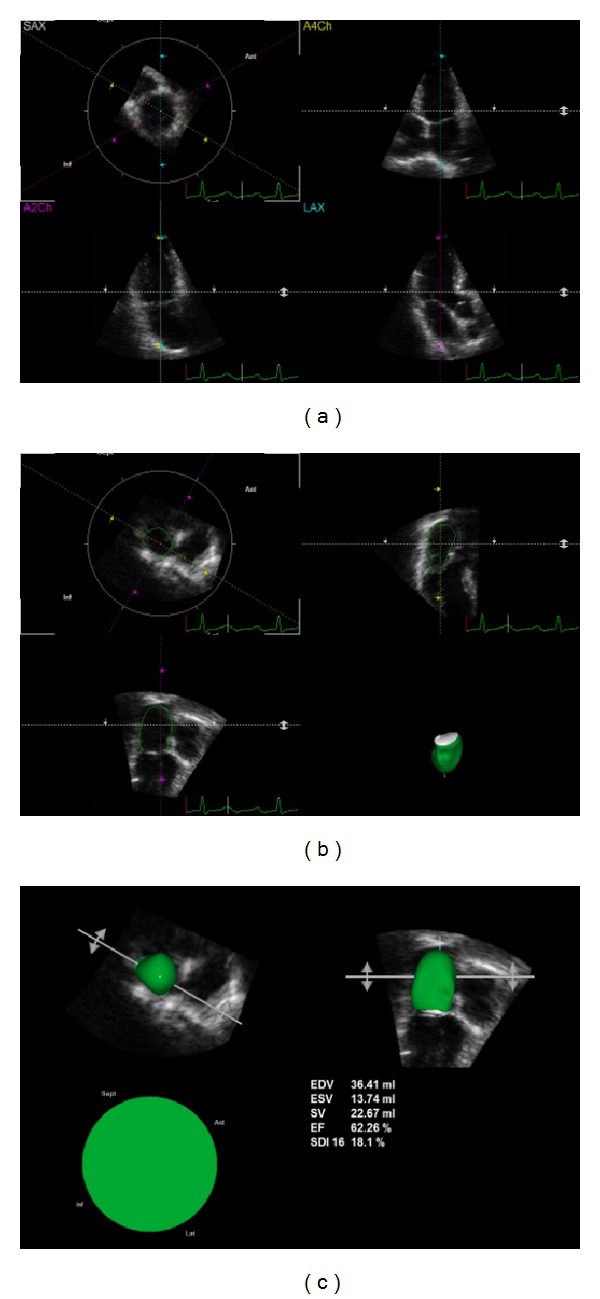
Left atrial volumes by TomTec 4D echocardiography. (a) After LA full-volume data acquisition, TomTec software displays LA in 3 different apical planes (4-chamber; 2-chamber, long-axis). (b) The endocardial border is surrounded on end-diastolic and end-systolic frames and, if needed, manually corrected. (c) Atrial maximum (max) and minimum (min) volumes are obtained using semiautomated border detection algorithm and displayed in numeric values and in 3D image (in green).

For TomTec 4D method, analysis of 3D images is performed surrounding endocardial border on end-diastolic and end-systolic frames on 3 different apical planes (4-chamber; 2-chamber, long-axis) and, if needed, manually corrected. The software analysis (TomTec) uses a semiautomated border detection algorithm [83] (Figure 8).

### 5.8. 3D Speckle Tracking

Three-dimensional (3D) speckle tracking echocardiography (3DS) has recently been developed, and its advantages for the determination of LV S [84–88] and LV synchrony [89] have been shown to overcome some limitations of speckle tracking imaging. The measurement of LA S by 2DS is dependent on image quality and suffers from errors due to the loss of some speckles that move out of the image plane (i.e., through-plane motion) [90]. LA myocardial fibers are arranged in both longitudinal and circumferential directions [91], and cardiac magnetic resonance imaging has demonstrated that LA fibrosis occurred heterogeneously in patients with AF [92]. Thus, it is likely that the assessment of longitudinal LA function by 2DS overlooks some LA dysfunction.

Recently, three-dimensional speckle tracking was used to examine the feasibility and reproducibility of 3DS for the determination of LA S and synchrony in healthy subjects and to investigate the effects of AF on LA parameters by 3DS. Although 3D LA S showed excellent reproducibility and appears to be beneficial compared with 2D LA strain for identifying patients with parossistic AF [7, 93], in the future, more validation studies will be required to investigate their capability in detecting dysfunctional LA regions. 3D S software packages offered by different vendors should be standardized, and population-based studies should be initiated to define normal reference values for 3D S global and regional S values. Finally, large-scale clinical studies will be required to assess whether 3D S values have any diagnostic or prognostic value in different clinical settings before these methods can effectively be applied into clinical use [94].

## 6. Conclusions

The LA plays a critical role in the clinical expression and prognosis of patients with heart disease. Noninvasive echocardiography is rapidly advancing toward a clinically feasible method of choice for the quantitative analysis of LA function.

Together with traditional parameters, such as LA diameter, area, and volume, strain and strain rate, by Doppler myocardial imaging and by 2D and 3D speckle tracking, are able to detect early functional remodelling before anatomical alterations occur, providing independent and additional prognostic information to conventional LA measurements.

## Figures and Tables

**Table 1:** 

Disease	LA function mainly impaired	Advantages	Extensive clinical data
Atrial fibrillation	Reservoir and conduit decreases; pump absent	Prediction of sinus rhythm maintenance after cardioversion	Yes

Mitral stenosis	Reservoir	Prediction of adverse events (AF, symptoms, hospitalization, thromboembolic events, valvular surgery, and percutaneous commissurotomy)	Yes

Mitral regurgitation	Reservoir	Prediction of AF, cardiac surgery, and LA fibrosis	Yes

Aortic stenosis	Pump	Prediction of adverse outcomes	No

Diastolic dysfunction	Conduit (early stage); pump and reservoir (late stage)	Elevated filling pressure	Yes

Dilated cardiomyopathy	Pump and reservoir	Responsivity to cardiac resincronization therapy	No

Diabetes mellitus	Pump, reservoir, and conduit	Early sign of atrial fibrosis	No

Hypertrophic cardiomyopathy	Reservoir	Prediction of AF and the onset of symptoms of heart failure	Yes

Amyloidosis	Reservoir	Early LA involvement in absence of the classic echocardiographic features	No

Hypertension	Reservoir	Prediction of myocardial involvement before LA enlargment and LV hypertrophy	Yes
